# Soft tissue evaluation of functional therapy in growing patients with class II malocclusion: mandibular advancement vs. twin block—a retrospective study

**DOI:** 10.3389/fdmed.2025.1581032

**Published:** 2025-09-02

**Authors:** L. Lugli, C. Pavoni, F. C. De Razza, F. Gazzani, S. Loberto, E. Cretella Lombardo, P. Cozza, R. Lione

**Affiliations:** ^1^ Department of Health Science, UniCamillus-Saint Camillus International Medical University, Rome, Italy; ^2^ Department of Oral and Maxillofacial Sciences, Sapienza University of Rome, Rome, Italy

**Keywords:** class II malocclusion, functional therapy, clear aligners, twin block, mandibular advancement

## Abstract

**Objective:**

The aim of this retrospective study was to assess the soft tissues changes resulting from Class II treatment with functional appliances (Twin Block vs. Mandibular Advancement), when compared to an untreated Class II control group.

**Materials and methods:**

The records of 45 Class II patients who underwent treatment with Twin Block (TB group: *n* = 22; mean age: 11.3 ± 1.4 years) or Mandibular Advancement (MA group: *n* = 23; mean age: 11.2 ± 1.3 years) were analyzed in comparison with a control sample of untreated Class II subjects (Untreated Control group = 24; mean age: 11.2 ± 1.1 years). The data were collected before treatment (T1) and at the conclusion of the functional therapy phase (T2). Cephalometric modifications were assessed among the three groups using ANOVA and Tukey's *post hoc* tests.

**Results:**

Significant improvements were observed for mandibular sulcus and the facial profile angle in the treated groups compared to the Untreated control group (facial profile angle: TB group: +5.67°; MA group: +6.34°; mandibular sulcus: TB group: +10.0°; MA group: 11.77°). The distance of Pogonion (Pg) to the True Vertical Line (TVL) exhibited significant differences among the three groups, with a more pronounced advancement of the soft tissue pogonion in the TB group (TVL-Pg': TB group: +3 mm; MA group: +0.9 mm; untreated control group: −1.6 mm).

**Conclusions:**

Treatment with removable functional devices (TB or MA) during puberty produced beneficial effects on the soft tissue profile. Both treated groups demonstrated a significant improvement in the Class II convex profile, accompanied by a less evident mandibular sulcus.

## 1 Introduction

Class II dento-skeletal malocclusion is often associated to a backward-positioned mandible (1–3). In growing patients, functional therapy encourages mandibular growth by repositioning the lower jaw forward, allowing the condyles to settle in a more anterior and inferior position within the glenoid fossa (2–6).

A key therapeutic goal of all functional appliances is to enhance facial aesthetics by decreasing the convexity of the profile while improving lip projection and closure (5).

Various functional devices, including Andersen's activator, Bionator, Frankel, Sanders, and Twin Block (TB) appliances, have been specifically developed to promote mandibular advancement and facilitate its forward repositioning, aiming to correct Class II skeletal imbalances (7).

The dentoskeletal effects of functional appliances have been widely studied in the literature, as well as their impact on soft tissues. Twin Block appliance demonstrated significant improvements in facial profile aesthetics and mandibular advancement when applied in patients at the pubertal growth peak (3, 8–13). In particular, Baysal et al. evaluated 25 patients (mean age: 12.9 ± 1.4 years) treated with the Twin Block appliance and reported a significant reduction in facial convexity and advancement of the lower lip, improving soft tissue profile (12). In addition, Varlık et al., in a prospective study on 28 growing patients (mean age: 12.2 ± 1.3 years), highlighted a significant increase in soft tissue pogonion projection and a reduction in upper lip protrusion following Twin Block therapy (13).

In recent years, with the spread of clear aligner therapy, Align Technology (San Jose, CA, USA) introduced the Mandibular Advancement (MA) feature. Precision wings built into the aligners, gradually posture the mandible forward.

Despite numerous studies evaluating the skeletal and dental effects of MA therapy (11–18), research on its impact on soft tissue changes remains limited (18–21).

Sabouni et al. reported a significant decrease in facial convexity and a marked increase in the nasolabial angle among 32 patients with an average age of 13 years old treated with the MA appliance, while changes in the chin angle were not significant (18).

In a retrospective study by Sadek et al. involving 15 growing patients (mean age: 11.6 ± 2.35 years), a significant reduction in both facial convexity and upper lip protrusion was observed (19).

In addition, Goje et al., examined 20 patients with a mean age of 13 years treated with the MA appliance and highlighted a significant improvement in the nasolabial angle and a not significant reduction of the chin (20).

However, only a recent study (21) directly compared the dentoskeletal and soft tissue changes in a group of 50 subjects (mean age: 11.98 ± 2.18 years) treated with MA vs. an untreated control group of patients with Class II malocclusion. The findings highlighted significant improvements in mandibular positioning and overall skeletal and dental changes, which contributed to better facial profiles and soft tissue adaptation.

To our best knowledge no studies have analyzed the esthetic outcomes comparing the single-step advancement of the TB appliance and the gradual advancement of the MA with clear aligners.

Therefore, the present retrospective study aimed to analyze the soft tissue modifications induced by Class II treatment with functional therapy (TB vs. MA) compared to an untreated Class II control group.

## 2 Materials and methods

This retrospective controlled study received approval from the Ethical Committee of the Hospital of Rome “Tor Vergata” (protocol no. 48/23). Written informed consent was obtained from all participants' parents.

The sample size was calculated considering an effect size for the primary outcome variable Facial profile angle (G'-SubN-Pg') of 1.0, an alpha value of 0.05 and a power of 0.80. At least 15 subjects were required for each group.

**Figure 1:**
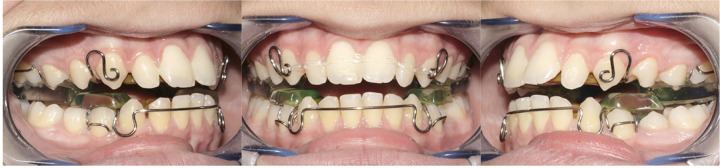
Frontal and lateral views of TB appliance.

Two groups of subjects with a Class II Malocclusion were selected from files of the Departments of Orthodontics at the Hospital of Rome “Tor Vergata” between January 1, 2019, and January 1, 2022. The first group (TB group) consisted of 22 patients (14 males, 8 females; mean age: 11.3 ± 1.4 years) treated with Twin Block appliance (Figure 1).

**Figure 2:**
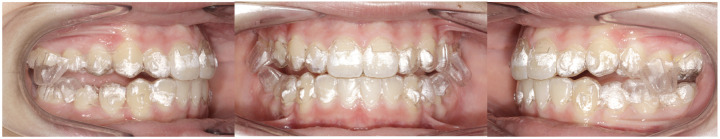
Frontal and lateral views of MA appliance.

The second group (MA group) was composed of 23 patients (11 males, 12 females; mean age: 11.2 ± 1.3 years) treated with clear aligners with MA feature appliance (Figure 2).

Inclusion criteria consisted of: Caucasian ethnicity, overjet greater than 5 mm, full Class II or end-to-end molar relationships, ANB angle greater than 4°, improvement in facial profile when the lower jaw was postured in a forward position and circumpubertal stage (stage 2 and 3) according to the Cervical Vertebral Maturation (CVM) at the start of treatment (22). CVM staging was performed by an expert examiner (RL).

Subjects with facial asymmetry, craniofacial syndromes, previous orthodontic treatment were excluded. No permanent teeth were congenitally missing or extracted before or during treatment.

Lateral cephalograms were available: before treatment (T1) and at the end of functional therapy (T2), before orthodontic therapy with both fixed appliance or the continuing phase with additional aligners. Functional treatment was discontinued after the achievement of Class I molar relationship.

24 untreated subjects with Class II division 1 malocclusion (Untreated Control group) were selected from the American Association of Orthodontists Foundation (AAOF) Craniofacial Growth Legacy Collection (http://www.aaoflegacycollection.org, Bolton–Brush Growth Study, Michigan Growth Study, Denver Growth Study, Oregon Growth Study, and Iowa Growth Study) and were matched with treated groups for age, skeletal development, dentition development, skeletal features and observation time intervals. The inclusion criteria of the control group were the same as those of the treated samples.

### 2.1 Cephalometric analysis

**Figure 3:**
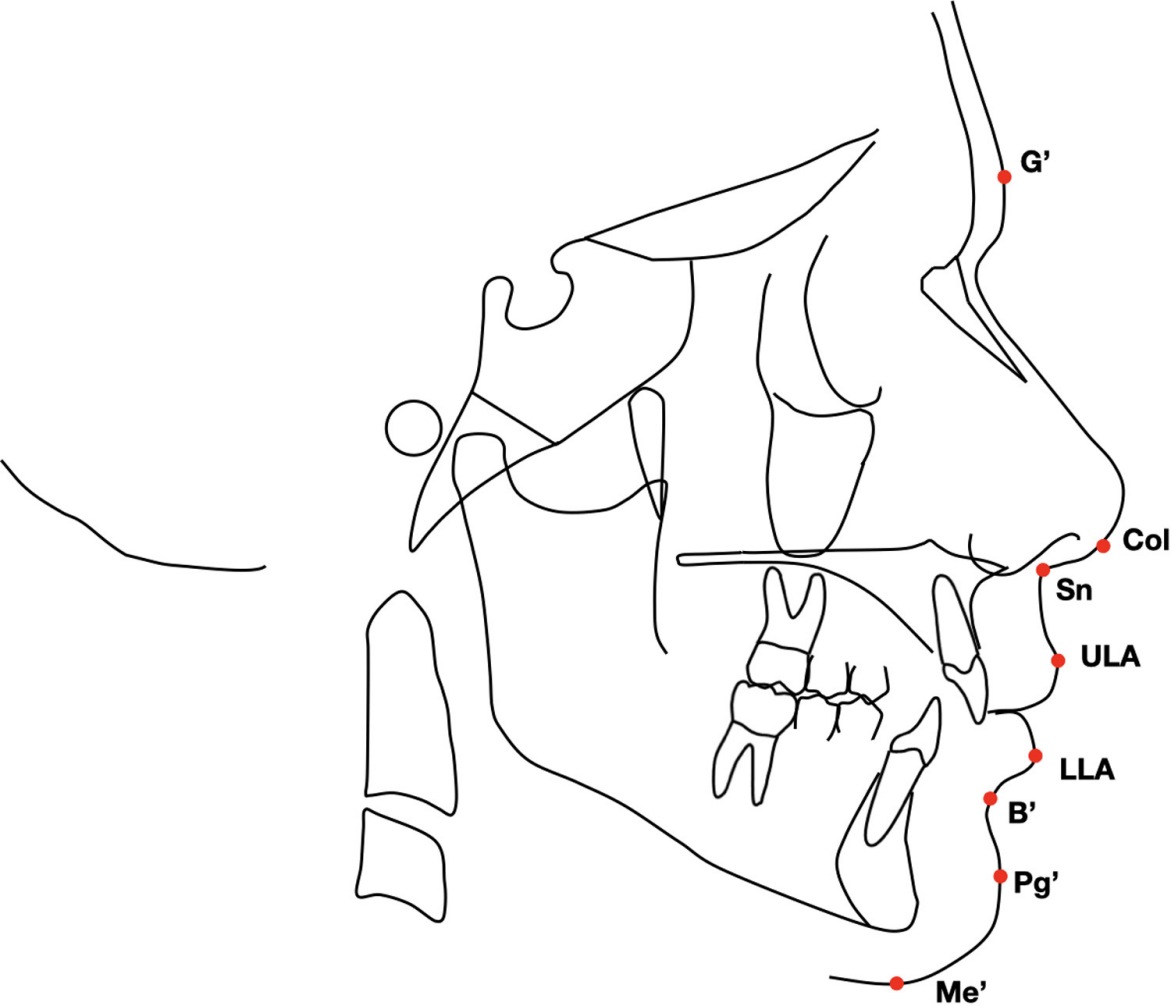
Soft tissue landmarks used in analysis.

Lateral cephalograms for both treated and untreated subjects were scanned at the same resolution (150 dpi). A customized digitization regimen and cephalometric analysis provided by Viewbox (version 3.0; dHAL Software, Kifissia, Greece) were used for all cephalograms selected. The customized soft tissue cephalometric analysis (Figure 3), containing measurements from the analysis of Bergman (23), generated six variables, three angular and two linear, and one percentage value. In addition to these soft tissue cephalometric traits, the distance from the soft tissue pogonion to the true vertical line (TVL) was measured using Arnett's analysis (24).

All the soft tissue cephalometric measurements are summarized in Table 1.

**Table 1:** Soft tissue cephalometric variables and their definitions.

Variables	Definition
Nasolabial angle (deg)	Angle formed by the intersection of upper lip anterior and columella at subnasale
Mandibular sulcus (deg)	Angle formed by the lower lip anterior, soft tissue B point, and soft tissue pogonion when the lips are in repose
Lower Lip protrusion (mm)	Perpendicular distance between lower lip anterior and the subnasale-pogonion line
Upper Lip protrusion (mm)	Perpendicular distance between upper lip anterior and the subnasale-pogonion line
Lower face %	Lower third of the face from subnasale to soft tissue menton, measured vertically and expressed as a percentage of the midface and lower face height, measured from soft tissue glabella vertically to soft tissue menton
Profile facial angle (deg)	Angle formed by connecting soft tissue glabella, subnasale, and soft tissue pogonion
Distance TVL-Pg’ (mm)	Distance from the soft tissue Pogonion to the true vertical line

**Figure 4:**
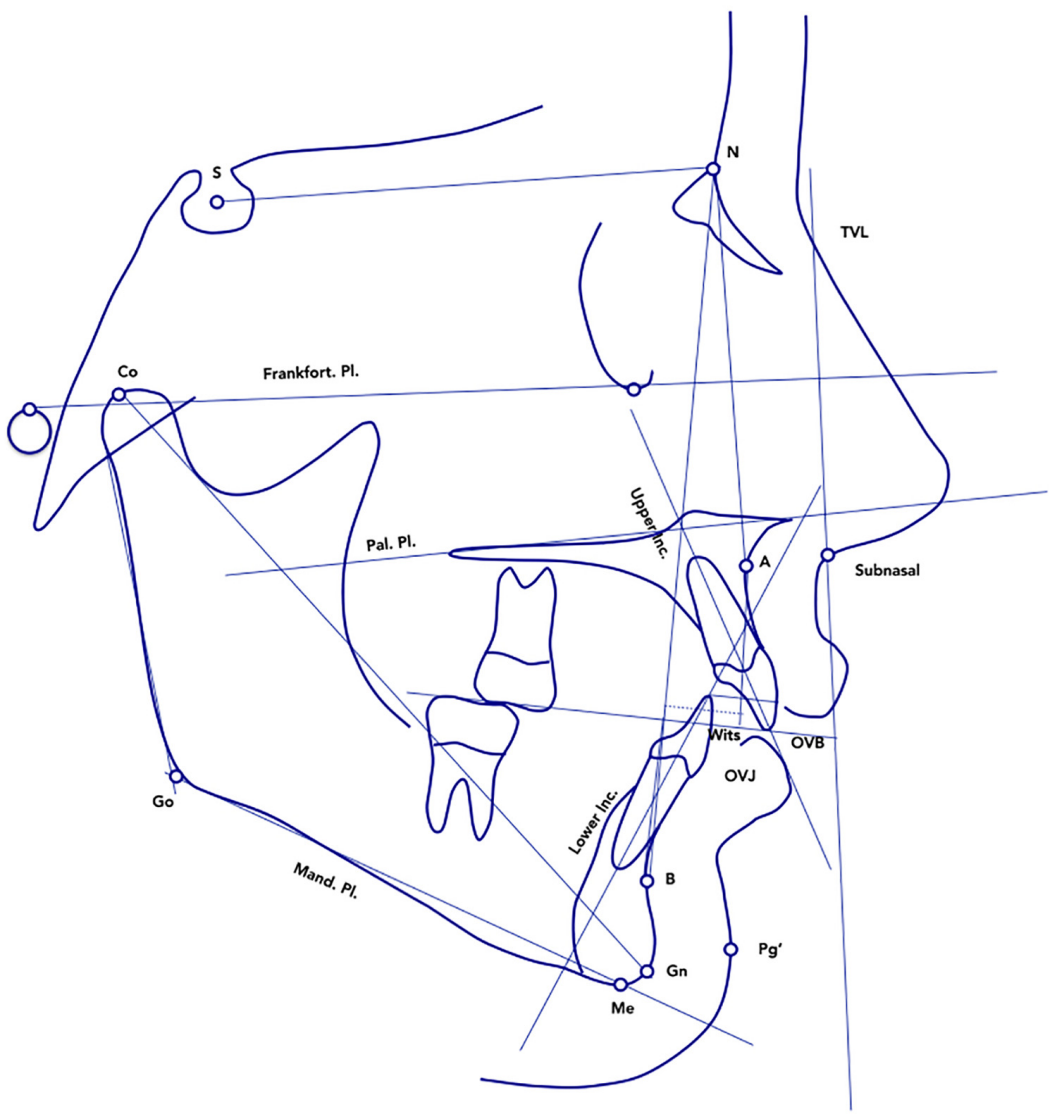
Cephalometric parameters measured at T1 and at T2.

Additional cephalometric variables were digitized for each patient at T1 and T2 in order to provide data on the dento-skeletal correction (Figure 4 and Table 2).

**Table 2:** Cephalometric variables and their definitions.

Variables	Definition
SNA (deg)	Angle between Sella, Nasion, and point A
SNB (deg)	Angle between Sella, Nasion, and point B
ANB (deg)	Angle between point A, Nasion, and point B
WITS appraisal (mm)	Distance between points A and B projected on the occlusal plane
Co-Gn (mm)	Mandibular length from Condylion to Gnathion
SN-Palatal plane (deg)	Angle between the Sella-Nasion line and the Palatal Plane
SN-Mandibular plane (deg)	Angle between the Sella-Nasion line and the Mandibular Plane
Palatal plane-Mandibular plane (deg)	Angle between the Palatal plane and the Mandibular plane
Co-Go-Me (deg)	Gonial angle formed by Condylion, Gonion, and Menton
Overjet (mm)	Horizontal distance between upper and lower incisors
Overbite (mm)	Vertical distance between upper and lower incisors
Upper incisor to palatal plane (°)	Inclination of the upper incisor relative to the palatal plane
Lower incisor to mandibular plane (°)	Inclination of the lower incisor relative to the mandibular plane

All lateral cephalograms at T1 and T2 were standardized to life size (0% enlargement).

### 2.2 Statistical analysis

The Chi-square test of independence was used to assess differences in gender distribution between the three groups.

Descriptive statistics and intergroup comparisons of cephalometric changes were performed using ANOVA and Tukey's *post hoc* tests at T1 (starting forms) and for the T2–T1 changes. When the variables were not normally distributed (Shapiro–Wilk test), Kruskal–Wallis with Dunn's *post hoc* tests were performed (25).

### 2.3 Method error

To determinate the method accuracy, one trained examiner (LL) with an experience of 4 years performed all the measurements on lateral cephalograms and 20 radiographs were retraced after an interval of approximately 2 weeks. Intra-observer reproducibility was assessed with the intraclass correlation coefficient (ICC), while for the assessment of the random error the method of moments' estimator (MME) was applied (26).

## 3 Results

The demographic data of the treated and the control groups are reported in Table 3.

**Table 3:** Demographics of the treated and control groups.

Group	Age at T1 (years)	Age at T2 (years)	T1-T2 interval (years)
	Mean SD	Mean SD	Mean SD
TB group (*n* = 22, 8 F, 14 M)	11.3 ± 1.4	12.8 ± 1.5	1.5 ± 2.0
MA group (*n* = 23, 12 F, 11 M)	11.2 ± 1.3	12.7 ± 1.2	1.5 ± 1.7
Untreated control group (*n* = 24, 12 F, 12 M)	11.2 ± 1.1	12.6 ± 1.3	1.4 ± 1.7

TB, twin block; MA, mandibular advancement; SD, standard deviation; F, females; M, males; ys, years.

No significant between-group differences were found either for chronologic age at T1 (*P* = 0.492), chronologic age at T2 (*P* = 0.896), chronologic age at T2-T1 interval (*P* = 0.545) and for gender distribution (*P* = 0.789). The chi-square test showed no statistically significant difference in gender distribution within the examined group (*p* = 0.49).

The intra-observer reproducibility, evaluated with the ICCs, indicated a high level of intraobserver agreement (ICCs varied between 0.897 and 0.999). As for the measurement errors they varied from 0.2° to 0.4° for the angular measurements and from 0.4 to 0.5 mm for the linear measurements.

No significant between-group differences were found in any of the variables at T1 (Table 4).

**Table 4:** Descriptive statistics and statistical comparisons of baseline characteristics. Analysis of variance (ANOVA) with Tukey's *post hoc* tests or ANOVA on ranks with Dunn's *post hoc* tests.

Variables	TB group (1) (*n* = 22)		MA group (2) (*n* = 23)		Untreated control group (3) (*n* = 24)		*P*	Multiple test comparisons								
	Mean	SD	Mean	SD	Mean	SD		1 vs. 2			1 vs. 3			2 vs. 3		
								Diff	*P*	95% Cl	Diff	*P*	95% Cl	Diff	*P*	95% Cl
G’-SubN-Pg (°)	139.9	1.9	139.6	2.1	140.6	3.0	0.5	–0.3	0.89	–1.7 to 1.23	–0.7	0.59	–1.7 to +1.2	–0.9	0.44	–1.4 to +1.4
Nasolabial (°)	129.3	11.7	128.8	11.7	128.3	9.7	0.9	–0.5	0.76	–8.4 to +7.4	–1.0	0.63	–8.8 to +6.7	–0.5	0.67	–8.2 to +7.1
Lower face (%)	51.6	1.5	51.4	1.6	50.9	2.2	0.6	0.02	0.56	–1.3 to 1.3	–0.9	0.72	–2.2 to +0.3	–0.9	0.88	–2.1 to +0.4
UpperLip-SubN/Pg’ (mm)	3.4	0.6	3.3	0.8	3.7	1.9	0.8	0.01	0.87	–0.7 to +0.7	–0.3	0.65	–1.0 to +0.4	–0.3	0.76	–1.0 to +0.4
LowerLip-SubN/Pg’ (mm)	1.9	0.6	1.7	0.7	1.9	2.6	0.5	–0.1	0.71	–1.3 to +1.0	0.1	0.58	–1.0 to +1.2	0.2	0.58	–0.9 to +1.3
Mandibular sulcus (°)	127.8	8.5	127.6	6.4	123.7	13.1	0.6	–0.2	0.82	–7.3 to +6.8	–4.1	0.88	–11.0 to +2.8	–3.9	0.32	–10.7 to +2.9
TVL-Pg’	–10.9	4.1	–8.9	4.6	–8.7	2.7	0.6	–2.0	0.55	–4.6 to +0.7	–2.2	0.78	–5.2 to +0.7	–0.2	0.25	–3.5 to +3.0

SD, standard deviation; Diff., differences; 95% CI, 95% confidence interval; *P*, *P* value; G, Gonion; SubN, Subnasale; Pg, Pogonion; Gn, Gnation; TVL, true vertical line; mm, millimetres; TB, twin block; MA, mandibular advancement; TVL, true vertical line; *P < 0.05; **P < 0.01; ***<0.001; NS, not significant.

The statistical comparisons of the T2–T1 changes between TB, MA and control groups (Table 5) showed significant modifications produced by functional therapy.

**Table 5:** Descriptive statistics and statistical comparisons of the T2–T1 changes. Analysis of variance (ANOVA) with Tukey's *post hoc* tests or ANOVA on ranks with Dunn's *post hoc* tests.

Variables	TB group (1) (***n* **= 22)		MA group (2) (***n* **= 23)		Untreated control group (3) (***n* **= 24)		***P***	Multiple test comparisons								
	Mean	SD	Mean	SD	Mean	SD		1 vs. 2			1 vs. 3			2 vs. 3		
								Diff	***P***	95% Cl	Diff	***P***	95% Cl	Diff	***P***	95% Cl
G’-SubN-Pg (°)	5.7	4.8	6.3	4.6	–0.6	3.7	**<0.001**	0.6	0.87	–2.4 to +3.8	–6.3	**<0.001**	–9.3 to –3.3	–6.9	**<0.001**	–9.9 to –3.9
Nasolabial (°)	–0.3	7.2	–0.3	7.1	0.3	10.3	0.72	0.0	0.75	–5.9 to +6.0	0.6	0.91	–5.2 to +6.5	0.6	0.74	–5.2 to +6.4
Lower face (%)	–0.4	2.0	–0.3	1.5	0.6	2.1	0.68	0.1	0.92	–0.3 to +2.6	1.1	0.78	–0.3 to +2.5	0.9	0.82	–1.4 to 1.3
UpperLip-SubN/Pg’ (mm)	–0.8	1.2	–0.5	1.8	–0.2	1.1	0.74	0.3	0.58	–-0.7 to +1.3	0.6	0.63	–0.4 to +1.6	0.3	0.91	–0.6 to +1.3
LowerLip-SubN/Pg’ (mm)	–0.5	0.7	–0.4	0.9	0.2	2.3	0.83	0.1	0.78	–0.9 to +1.2	0.7	0.82	–0.4 to 1.8	0.6	0.78	–0.5 to +1.6
Mandibular sulcus (°)	10.0	2.1	11.8	10.0	0.8	10.8	**<0.001**	1.8	0.68	–4.5 to +7.9	–9.2	**<0.001**	–15.3 to –3.1	–10.0	**<0.001**	–17.0 to –4.9
TVL-Pg’ (mm)	3.0	2.0	0.9	3.7	–1.6	3.3	**<0.001**	–2.1	**0.041**	0.2 to +4.0	4.6	**<0.001**	+2.5 to +6.7	2.5	**0.032**	0.2–4.8

SD, standard deviation; Diff., differences; 95% CI, 95% confidence interval; *P*, *P* value; G, Gonion; SubN, Subnasale; Pg, Pogonion; Gn, Gnation; TVL, true vertical line; mm, millimetres; TB, twin block; MA, mandibular advancement; TVL, true vertical line; *P < 0.05; **P < 0.01; ***<0.001; NS, not significant.

Both the TB and MA groups showed a significant and clinically relevant increase in the profile facial angle (G'-SubN-Pg') compared to the untreated control group (TB: +5.7°; MA: +6.3°; Untreated Control group: −0.6°). Additionally, for the mandibular sulcus parameter, the TB and MA groups demonstrated significant increases (TB: +10°; MA: +11.8°) compared to the control group (Untreated Control group: 0.8°).

The increase in the distance of Pg to TVL from T1 to T2 showed a statistically significant difference across the three groups, with a greater advancement of the soft tissue pogonion in the TB group compared to both the MA group and the Untreated Control group (TB group: +3 ± 2 mm; MA group: +0.9 ± 3.7 mm; Untreated Control group: −1.6 ± 3.3 mm).

During the T2–T1 interval, the TB and MA groups showed significant dento-skeletal changes compared to controls (Supplementary Table S1), including a reduction in the ANB angle (TB: −1.5°; MA: −1.5°; Untreated Control group: +0.2°) and an increase in Co-Gn values (TB: +8.4 mm; MA: +8.3 mm). Both groups also demonstrated a greater reduction in the palatal plane-mandibular plane angle and improvements in overjet and vertical overbite values.

## 4 Discussion

Functional therapy affects skeletal and dentoalveolar structures as well as soft tissue organization, leading to a more balanced and aesthetically pleasing facial profile in relation to craniofacial structures (26–30).

The present study aimed to assess the impact of functional therapy (TB vs. MA) on the soft tissue facial profile in growing Class II patients, when compared with untreated Class II control group.

One of the main strengths of our study was the inclusion of a control group. This allowed for a direct comparison of the outcomes observed in the treatment group against a baseline, enhancing the validity of our results on soft tissue changes. The control group was carefully selected to minimize confounding variables, such as age, sex, and initial craniofacial morphology, ensuring that the observed differences can be attributed primarily to the treatment.

As reported by many authors treatment with removable functional appliances produced significant skeletal long-term changes when it begins at puberty (3, 31). Therefore, one of the inclusion criteria for subject selection was the circumpubertal stage of cervical vertebral maturation at the start of treatment (CVM2-CVM3). Moreover, the study compared two removable functional appliances, TB and MA, which are based on the same mechanism of the inclined planes that posture the jaw to an advanced forced position.

Despite soft tissue changes induced by TB have been evaluated in the literature (12, 13, 31–35), data concerning aesthetic improvements and soft tissue modifications produced by MA are limited.

Overall, T2–T1 changes showed a significantly more harmonious facial profile in treated groups (TB and MA) compared to the untreated control group.

Profile facial angle significantly increased in treated groups with respect to the control group (TB group = +5.67° ± 4.76°; MA group: +6.34° ± 4.58°; untreated control group: −0.64° ± 3.67°). Our results are consistent with those reported in the literature, which highlighted a reduction in facial convexity in patients treated with MA (18, 21).

For the mandibular sulcus parameter, statistically significant differences were observed in the TB and MA groups compared to the untreated control group, with an increase of 10° in the TB group and 11.77° in the MA group. The straightening of the mandibular sulcus appears to be primarily a result of mandibular advancement.

To the best of our knowledge, no previous study has specifically analyzed this parameter. Our findings also revealed a statistically significant difference among the TB, MA, and control groups concerning chin advancement, assessed by measuring the distance of the soft tissue pogonion from the true vertical line (TVL). In this study, the TB appliance appeared to be more effective in promoting chin advancement and, consequently, in improving the facial profile. This result could be attributed to the different mandibular advancement mechanisms applied, specifically the one-step advancement with the TB appliance vs. the progressive advancement with the MA feature.

In the literature there are very conflicting opinions on which is the most effective advancement protocol. Nowadays, there is greater scientific evidence in favor of incremental mandibular advancement in terms of mandibular response and increase in mandibular length (15). It is interesting to note that on the basis of our results, the different advancement protocol applied by these two devices did not produce differences in the improvement of soft tissues.

All the profile modifications were mainly determined during the functional active treatment phase and were supported by dento-skeletal changes, as detailed in the Supplementary Table S1.

The results obtained should be supported and corroborated by further investigations to be conducted by means of long-term observation. The limitations of the present study are the use of historical controls, and the relatively small sample size. Ideally, a contemporary control sample should have been used because it has been shown that our face and its changes are influenced by secular trends (36). However, it would be unethical to leave a Class II sample without any treatment in the long term. Despite these limitations, the study design allowed for meaningful comparisons, and the sample size was justified through statistical computation to achieve sufficient power.

## 5 Conclusions

Treatment with removable functional appliances (TB or MA) at puberty induced a reduction in the convexity of the soft tissue facial profile. All modifications observed were mainly sustained by significant sagittal skeletal changes. In the treated groups, there was a notable improvement in the normalization of the Class II convex profile, accompanied by a more harmonious lip position, largely attributed to the straightening of the mandibular sulcus. Based on these results, both TB and MA appliances can be considered effective options for improving the soft tissue profile in growing Class II patients with mandibular deficiency. However, it remains essential to carefully assess the optimal treatment timing to maximize treatment effectiveness and stability.

## Data Availability

The raw data supporting the conclusions of this article will be made available by the authors, without undue reservation.
