# Supplementary material for: Soft tissue evaluation of functional therapy in growing patients with class II malocclusion: mandibular advancement vs. twin block—a retrospective study
Source: Front Dent Med. 2025 Sep 2;6:1581032. doi: 10.3389/fdmed.2025.1581032 (PMC12436345; doi:10.3389/fdmed.2025.1581032)
Supplement: Supplementary file 1 [file Table1.doc]

**Supplementary Table 1.** Descriptive statistics and statistical comparisons of the T2-T1 changes (ANOVA with Tukey’s post-hoc tests or ANOVA on Ranks with Dunn’s post-hoc tests)

| **Variables** | **TB Group (1)  (n=22)** | | **MA Group (2) (n=23)** | | **Control Group (3)**  **(n=24)** | | **P** | **Multiple test comparisons** | | | | | | | | |
| --- | --- | --- | --- | --- | --- | --- | --- | --- | --- | --- | --- | --- | --- | --- | --- | --- |
|  | **Mean** | **SD** | **Mean** | **SD** | **Mean** | **SD** |  | **1vs2** | | | **1vs3** | | | **2vs3** | | |
| **Sagittal Skeletal** |  |  |  |  |  |  |  | **Diff.** | **P** | **95% CI** | **Diff.** | **P** | **95% CI** | **Diff.** | **P** | **95% CI** |
| SNA (deg) | 0.1 | 1.7 | -0.4 | 2.6 | -0.7 | 1.5 | NS | 0.5 | NS | -0.8 1.8 | 0.8 | NS | -0.7 2.2 | 0.3 | NS | -1.2 1.9 |
| SNB (deg) | 1.6 | 1.4 | 1.4 | 3.2 | 0.4 | 1.1 | NS | 0.2 | NS | 1.1 1.6 | 1.2 | NS | -0.3 2.7 | 1.0 | NS | -0.7 2.6 |
| ANB (deg) | -1.5 | 1.4 | -1.5 | 1.5 | 0.2 | 0.3 | ******* | 0.0 | NS | -0.9 0.8 | **-1.7** | ******* | -2.6 -0.7 | **-1.7** | ******* | -2.6 -0.6 |
| Wits (mm) | -1.1 | 2.9 | -0.8 | 2.4 | 0.4 | 2.3 | NS | -0.3 | NS | -2.0 1.4 | -1.5 | NS | -3.5 0.4 | -1.2 | NS | -3.3 0.9 |
| Co-Gn (mm) | 8.4 | 2.0 | 8.3 | 3.1 | 3.3 | 1.2 | ******* | 0.1 | NS |  | **5.1** | ******* |  | **5.0** | ******* |  |
| **Vertical Skeletal** |  | | | | | | | | | | | | | | | |
| SN-Pal. Pl. (deg) | -0.4 | 1.9 | -0.3 | 3.6 | 0.2 | 1.2 | NS | -0.2 |  |  | -0.1 |  |  | 0.1 |  |  |
| SN-Mand. Pl. (deg) | -0.8 | 1.7 | -1.7 | 2.0 | 0.9 | 1.1 | NS | -0.1 | NS | -1.8 1.4 | -0.6 | NS | -2.5 1.1 | -0.5 | NS | -2.5 1.5 |
| Pal. Pl. - Mand. Pl. (deg) | -0.4 | 2.5 | -0.5 | 3.1 | 0.6 | 0.8 | ******* | 0.9 | NS | -0.2 2.0 | **-1.7** | ****** | -2.9 -0.4 | **-2.6** | ******* | -3.9 -1.2 |
| CoGoMe (deg) | -3.7 | 1.9 | -2.6 | 2.4 | 0.0 | 0.9 | 0.389 | 0.1 | NS | -1.5 1.7 | -1.0 | NS | -2.8 0.9 | -1.1 | NS | -3.0 0.9 |
| **Dentoalveolar** |  | | | | | | | | | | | | | | | |
| Overjet (mm) | -1.6 | 4.8 | -0.5 | 7.0 | 0.0 | 0.0 | ******* | -1.1 | NS | -2.3 0.2 | **-3.7** | ******* | -5.0 -2.2 | **-2.6** | ******* | -4.1 -1.0 |
| Overbite (mm) | 1.1 | 4.3 | -1.0 | 7.9 | 0.7 | 1.6 | ******* | -0.1 | NS | -1.1 0.9 | **-2.0** | ******* | -3.1 -0.9 | **-1.9** | ******* | -3.1 -0.7 |
| Upper Inc.- Pal. Pl. (deg) | 0.1 | 1.7 | -0.4 | 2.6 | -0.7 | 1.5 | NS | -1.1 |  |  | -1.6 |  |  | -0.5 |  |  |
| Lower Inc.- Mand. Pl. (deg) | 1.6 | 1.4 | 1.4 | 3.2 | 0.4 | 1.1 | NS | 2.1 | NS | -1.4 5.6 | 0.4 | NS | -3.5 4.3 | -1.7 | NS | -6.0 2.6 |

SD = standard deviation; Diff .= differences, 95% CI = 95% confidence interval; P = P value; G = Gonion; SubN = Subnasale; Pg = Pogonion; Gn = Gnation; TVL=True Vertical Line; mm = millimetres; TB = Twin Block; MA = Mandibular Advancement;; *P < 0.05; **P < 0.01; ***<0.001; NS=not significant
